# The impact of combined administration of surfactant and intratracheal budesonide compared to surfactant alone on bronchopulmonary dysplasia (BPD) and mortality rate in preterm infants with respiratory distress syndrome: a single-blind randomized clinical trial

**DOI:** 10.1186/s12887-024-04736-9

**Published:** 2024-04-20

**Authors:** Asghar Marzban, Samira Mokhtari, Pouria Tavakkolian, Reza Mansouri, Nahid Jafari, Azam Maleki

**Affiliations:** 1 Dept. of neonatology, School of Medicine, Musavi hospital, Zanjan university of medical sciences, Zanjan, Iran; 2 Student Research Center, Zanjan University of Medical Sciences, Zanjan, Iran; 3 Department of Hematology & Medical Oncology, Vali-e-Asr Hospital, Zanjan University of Medical Sciences, Zanjan, Iran; 4 Social Determinants of Health Research Center, Health and Metabolic Diseases Research Institute, Zanjan University of Medical Sciences, Azadi Square, Jomhori Eslami St, Zanjan, 4515613191 Iran

**Keywords:** Respiratory distress syndrome, Premature neonate, Budesonide, Surfactant

## Abstract

**Background:**

Respiratory distress syndrome (RDS) is one of the most important and common disorders among premature infants.

**Objective:**

This study aimed to compare the effect of the combination of surfactant and budesonide with surfactant alone on Bronchopulmonary dysplasia (BPD) and mortality rate among premature infants with RDS.

**Method:**

An outcome assessor-blind randomized clinical trial was conducted on 134 premature infants with RDS who were born in Ayatollah Mousavi Hospital, Zanjan, Iran in 2021. The covariate adaptive randomization method was utilized to allocate participants into two groups (surfactant alone and a combination of surfactant and budesonide). The primary outcomes were BPD and Mortality rate from admission to hospital discharge. The data in this study were analyzed using SPSS software version 18.

**Results:**

Overall the comparison of mortality rate and BPD between the two groups did not show a significant difference(*p* > 0.05). The subgroup results showed that administering surfactant with budesonide to infants under 30 weeks of age significantly reduced the number of deaths compared to using surfactant alone (5 vs. 17). Similar positive effects were observed for the occurrence of Pulmonary Hemorrhage, the need for a second dose of surfactant, oxygen index, mean blood pressure and mean arterial pressure (MAP) in infants under 34 weeks of age compared to more than 34 weeks (*p* < 0.05).

**Conclusion:**

These findings suggest that the combination therapy of surfactant and budesonide may be beneficial, particularly in preterm infants with less than 34 weeks gestational age and 1500 birth weight. However, further studies with larger sample sizes and longer follow-up periods are needed to confirm these results and assess long-term outcomes.

**Trial registration:**

The study was registered at the Iranian Registry of Clinical Trials website under the code IRCT20201222049802N1. https://en.irct.ir/user/trial/48117/view.

**Registration date:**

28/02/2021.

**Public repository: Data set:**

This research data set link is displayed on the Zanjan-Iran Medical Sciences website: https://repository.zums.ac.ir/cgi/users/login? target=https%3 A%2 F/repository.zums.ac.ir/id/eprint.

## Introduction

Respiratory Distress Syndrome (RDS) is a leading cause of morbidity and mortality in premature infants. It occurs when there are defects or delays in the production and secretion of surfactant, which leads to worsening symptoms within a few days if not treated with exogenous surfactant replacement. Premature infants are particularly susceptible to respiratory failure due to surfactant deficiency because their lungs are not fully developed. The inadequate surfactant levels result in alveolar collapse, reduced lung compliance, and decreased Functional Residual Capacity (FRC). These lung injuries also increase the risk of developing BPD [1]. BPD is defined as receiving any respiratory or ventilatory support or supplemental oxygen at 36 weeks postmenstrual age. BPD is a prevalent, intricate, and fascinating condition within the field of perinatal medicine [2].

Some infants with RDS may experience rapid respiratory distress, requiring increased oxygen supplementation, and may need continuous positive airway pressure (CPAP) or mechanical ventilation shortly after birth [1]. RDS has a slightly higher incidence in males compared to females. The major risk factors for RDS include prematurity, lower gestational age, and low birth weight. Infants born prematurely are at a higher risk due to the immaturity of their lungs and insufficient surfactant production. Additionally, maternal diabetes and perinatal hypoxia-ischemia are also considered risk factors for developing RDS. These factors contribute to the likelihood of experiencing respiratory distress and the need for interventions such as surfactant replacement therapy [3].

Over the past two decades, the administration of intratracheal surfactant has proven to be the most effective therapeutic intervention for treating Respiratory Distress Syndrome (RDS). This treatment has significantly reduced the occurrence of pneumothorax and improved overall survival rates. By reducing complications associated with RDS in preterm infants, surfactant treatment has played a crucial role in decreasing mortality rates, especially among those who require mechanical ventilation. The advancements in surfactant therapy have had a significant positive impact on the outcomes of preterm infants with RDS [4]. Postpartum steroid use has been shown to decrease the severity of BPD in infants. However, it is important to note that high doses of steroids can potentially result in developmental and neurological defects [5].

A combination of inhaled steroids and surfactants has recently been used in some studies [6, 7]. Preliminary data suggest that the combination of inhaled steroids and surfactants is safe and associated with a reduced risk of bronchopulmonary dysplasia or death in very low birth weight infants [6, 8]. In a study comparing the concomitant administration of surfactant and intratracheal budesonide with surfactant alone in preterm infants with RDS, it was found that the combination of budesonide and surfactant led to improved pulmonary status and reduced mortality and morbidity due to chronic lung disease, specifically BPD [7]. However, it is important to note that conflicting results have also been reported in other studies [9, 10]. The studies primarily focused on very premature babies. There is limited information available regarding the effects of this treatment on early and late preterm infants. Including late preterm infants in our study population allows us to assess the efficacy and safety of the combined treatment approach across a wider spectrum of preterm infants, providing valuable information for clinicians when making treatment decisions. Therefore, further research and investigation are needed to fully understand the potential benefits and effect of using intratracheal budesonide in combination with surfactant therapy on BPD and mortality rates in preterm infants with RDS.

## Method

### Study design and setting

An outcome assessor-blind randomized clinical trial was conducted on 134 premature infants with moderate to severe RDS who were born at a tertiary-level Neonatal Intensive Care Unit (NICU) in Ayatollah Mousavi Hospital, Zanjan, Iran in 2021. The study was registered at the Iranian Registry of Clinical Trials website under the code IRCT20201222049802N1. The date of protocol registration was 28/02/2021.

### Inclusion and exclusion criteria

Infants exhibiting the common clinical indications of respiratory distress syndrome, such as grunting, retraction of the intercostal, subcostal, and substernal areas, nasal flaring, cyanosis, increased oxygen demand, and a respiratory rate exceeding 60 breaths per minute, along with typical radiological findings of RDS including a diffuse reticulonodular pattern, classic Ground-Glass appearance in both lungs and a significant increase in air bronchograms shortly after birth, were categorized based on severity using the Downes scoring system [11]. Infants falling into the Moderate or Severe group were included in the study. Infants meeting the following criteria were included in the study: gestational age less than 37 weeks, birth weight less than 2500 g, the requirement for mechanical ventilation within 4 h after birth, requirement for Fio2 greater than 30% for infants with a gestational age less than 28 weeks, and greater than 40% for infants with a gestational age above 28 weeks, surfactant prescription candidate with expert doctor’s opinion.

Infants were excluded from the study if they met any of the following criteria: Weight less than 700 g, Severe congenital anomalies, Fatal cardiopulmonary disease, Other causes of respiratory distress, such as congenital diaphragmatic hernia, that could be identified through x-ray or echocardiography.

Neonates who had the classic clinical criteria of respiratory distress syndrome as well as typical radiological findings of respiratory distress syndrome and according to the severity rating table Downs criteria were in two moderate and severe groups, as well as neonates who were under non-invasive ventilation and non-invasive ventilation failure were also included in this study.

Non-invasive ventilation failure was defined as one of multiple parameters: [1] pH of 7.25 or less [2], increased PaCO2 [3], increased FiO2 requirement [4], need for an NPSIMV rate greater than 20/min [5], need for a peak inflation pressure on NPSIMV of 20 cm H2O or more [6], need for PEEP on NPSIMV of 8 cm H2O or more, or [7] severe apnea. The type of ventilator used for all neonates in the two groups was: the Maquet servo-i ventilator.

The type of non-invasive support was a nasal mask or nasal prongs with the infant flow driver continuous-flow NCPAP system.

### Outcomes

#### The primary outcome

The primary outcomes were BPD at 14 days after the start of the study and Mortality rate (from admission to hospital discharge). The diagnosis of BPD was based on the clinical evaluation (such as breathing much faster than usual, the bluish discoloration around the mouth or lips, pulling in of the skin between the ribs, below the chest or at the bottom of the neck just above the chest), and continued need for supplemental oxygen.

#### The secondary outcomes

The secondary outcomes assessed in the study included the duration of mechanical ventilation during the first three days of hospitalization, the need for an additional dose of CUROSURF surfactant during the same period, the oxygen index measured on the first and third day of treatment, the MAP rate on the first and third day of treatment, the length of hospitalization at the time of discharge, the incidence of intraventricular and cerebral hemorrhage, the incidence of pneumothorax and pulmonary hemorrhage from patient admission to hospital discharge, and the blood sugar levels and mean blood pressure measured on the first and third day of treatment. These secondary outcomes were evaluated to assess various aspects of neonatal health and treatment response throughout the hospitalization period.

#### Sample size

The sample size was determined using G power software from Apponic Heinrich-Heine-University, Germany, taking into account the variable of BPD and mortality as reported in the study by Gharehbaghi et al. [12]. The parameters considered were p1 = 0.31, p2 = 0.59, α error probability = 0.05, and power (1-β error probability) = 0.85. Initially, the calculated sample size was 61 individuals for each group. The sample size was calculated based on the mortality variable of about 11 people in each group. In this study, the largest number, 61 people, was selected as the sample size. However, after accounting for a 10% dropout rate, the sample size was increased to 67 individuals for each group.

#### Procedure

The covariate adaptive randomization method was utilized in this study to allocate participants into two groups [13]. The researchers identified gestational age and birth weight as important factors that needed to be balanced across groups to mitigate potential confounding effects. To achieve this, a randomization algorithm was employed that took into account the values of these covariates for each participant. The algorithm adaptively allocated participants to treatment groups, ensuring a balanced distribution of gestational age and birth weight across the groups. In this study a random sequence of number was created using random table numbering by a person did not involve in the research term. The created numbers were recorded on the card, and the cards were placed in the letter envelopes. As soon as the eligible participants entered the study, one of the envelopes was opened in order, revealing the participant’s allocated group.

In this study the intervention implemented by the second author. Due to the conditions of the infants, the person who involved in treatment was not blind instead of the outcome assessor was blind.

In the control group, neonates received intratracheal CUROSURF surfactant alone at a dose of 2.5 cc/kg. In the intervention group, neonates received the same dose of CUROSURF surfactant combined with intratracheal administration of budesonide at a dose of 0.25 mg or 1 cc/kg. Decisions to continue managing RDS treatment in all patients were made based on the standard protocol and guidelines of the NICU [14]. Curosurf is a natural surfactant. each vial of Curosurf (Poractant alfa) used in this trial 3 cc instillation suspension contains 240 mg phospholipid fraction from Porcine lung. (Made by Chiesie Pharma company of Italy). The recommended starting dose is 100–200 mg/kg (1.25- 2.5 ml/kg), additional doses of 100 mg/kg (1.25 ml/kg), each at about 6-12hourly intervals may also be administered if needed. Pulmicort inhaler solution used in this trial is a 2 cc suspension with 0.25 mg/ml concentration containing Budesonide. (Made by Swedish manufacture ASTRAZENECA which is imported by Cobel Daru company). The vials should be warmed to room temperature by holding it in the hand for a few minutes, before use, and gently turned upside down a few times, without shaking, in order to obtain a uniform suspension. The suspensions should be withdrawn from the vials using a sterile needle and syringe and mixed gently. A suitable tube should then be used to instill Curosurf with Budesonide into the lung directly into the lower trachea by passing a catheter through the tube after intubation of the patient. In this the outcome assessment researchers were blinded to the treatment groups.

### Statistical analysis

The data in this study were analyzed using SPSS software version 18. The normality of the data was assessed using the Kolmogorov-Smirnov test, and it was determined that the data had a normal distribution. The analysis of the data was conducted using the Chi-square test, Fisher Exact test, One-way ANOVA, and the Independent t-test. A significance level of 0.05 was used to interpret the results. The subgroup results were done based on the birth weight and gestational age. The Analysis of covariance (ANCOVA) was used for the comparison of mean (SD) secondary outcome between two groups by adjusting the baseline variables (first day MAP,” “first-day oxygen index,” “first-day blood sugar,” and “first day mean blood pressure).

## Results

### Baseline data

**Fig. 1:**
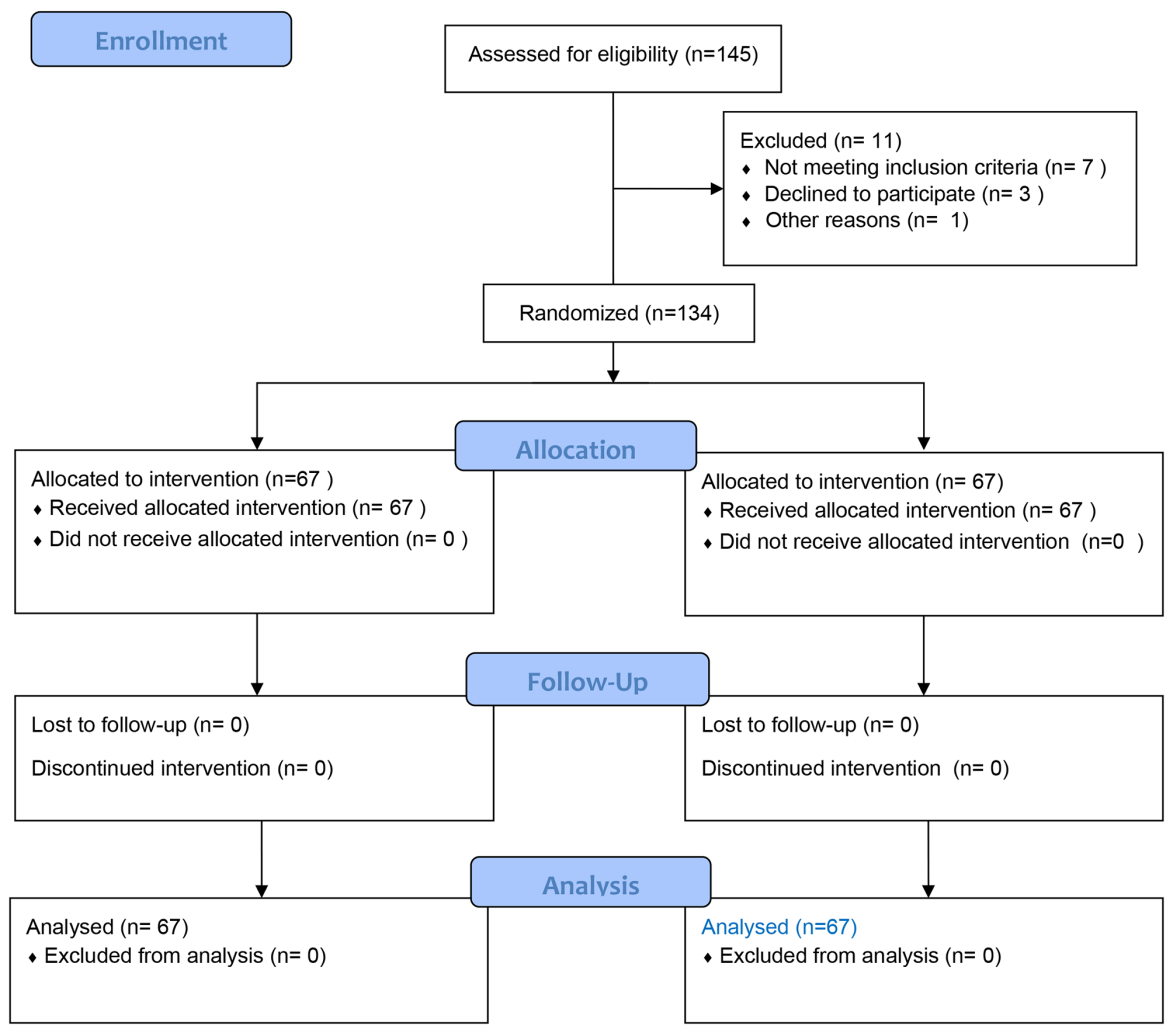
CONSORT flow diagram

In this study, a total of 145 infants were initially assessed based on eligibility criteria. Out of these, 134 infants met the inclusion criteria and were divided into two groups, each consisting of 67 individuals. The analysis and results of this study were based on these 134 participants, and there were no dropouts or individuals who did not complete the study. The process of selection of participants was shown in the CONSORT flow diagram (Fig. 1).

Table 1 presents the comparison of various demographic characteristics between the two groups. The result of independent t-test showed that the differences of gestational age (in weeks), birth weight (in grams), and maternal age (in years) were not statistically significant between two groups (*p* > 0.05).

**Table 1:** the comparison of demographic characteristics of infants between the two groups

Variables		Surfactant group (*n* = 67)	Surfactant + budesonide group (*n* = 67)
Gestational age (week)	Mean (SD)	31 ± 2.96	31.66 ± 2.84
Birth weight (gr)	Mean (SD)	1465.45 ± 520.88	1584.55 ± 505.02
Maternal age (year)	Mean (SD)	29.97 ± 6.45	29.12 ± 4.86
Delivery mode	NVD (n %)	19(28.4%)	18(26.9%)
	CS (n %)	48(71.6%)	49(73.1%)
Sex	Male (n %)	40(59.7%)	29(43.3%)
	Female (n %)	27(40.3%)	38(56.7%)
Maternal medical problems	Yes	22(32.8%)	19(28.4%)
	No	45(67.2%)	48(71.6%)
Antenatal drug	No	32(47.8%)	33(49.3%)
	Betamethasone	11(16.4%)	17(25.4%)
	Levothyroxine	3(4.5%)	5(7.5%)
	Combination	21(31.3%)	12(17.9%)
Gestational age (week)	< 30	24(35.8%)	16(23.9%)
	30–34	39(58.2%)	32(47.8%)
	> 34	12(17.9%)	11(16.4%)
Birth weight (gr)	< 1500	30(44.8)	40(59.7)
	1500–2000	22(32.8)	13(19.4)
	> 2000	15(22.4)	14(20.9)

The result of chi-square test showed that the frequency of delivery mode, sex, maternal medical problems, and maternal antenatal drug use did not have significant differences between two groups (*p* > 0.05). (Table 1).

### The primary outcome results

Overall, the comparison of mortality rate and BPD between the two groups did not show a significant difference. However, the number of cases of mortality (10 vs. 19) and BPD (9 vs. 10) in the group receiving combined surfactant and budesonide was lower than in the surfactant alone group (*p* > 0.05). In this study bronchopulmonary dysplasia (BPD) was followed up 14 days. In both groups, the duration of oxygen therapy in patients with BPD was 14 days. (Table 2).

**Table 2:** the comparison of primary and secondary outcome between two groups

Variables	Surfactant Group (*n* = 67)	Surfactant + Budesonide Group (*n* = 67)	*P* value*	Variables		Surfactant Group (*n* = 67)	Surfactant + Budesonide Group (*n* = 67)	*P* value**
	Mean (SD)	Mean (SD)				Frequency (%)	Frequency (%)	
Ventilation use length (day)	4.37 ± 2.81	2.85 ± 3.64	**0.008** T value= -2.70	Death ^†^	Yes	19(28.4)	10(14.9)	0.059 Chi-square values = 3.56 ***RR = 0.63, CI95% (0.37, 1.09)
	Mean diff = − 1.62, CI 95% (-2.63, − 0.41)							
Hospital stay (day)	16.21 ± 12.40	16.15 ± 15	0.679 T value= -0.02		No	48(71.6)	57(85.1)	
	Mean diff= -0.06, CI 95% (-4.76, 4.64)							
First -day- MAP (mmHg)	16.81 ± 1.64	15.48 ± 2.43	**0.001** T value= -3.70	BPD ^†^	Yes	10 (14.9)	8 (11.9)	0.612 Chi-square values = 0.257 RR = 0.87, CI95% (0.50, 1.50)
	Mean diff= -1.32, CI 95% (-2.03, -0.61)							
Third -day – MAP (mmHg)	11.05 ± 4.29	7.73 ± 2.86	**0.001** T value= -5.22		No	57(85.1)	59(88.1)	
	Mean diff= -3.31, CI 95% (-4.57, -2.06)							
First -day- oxygen index (%)	15.09 ± 7.24	12.70 ± 7.01	0.055 T value= -1.94	Pulmonary Hemorrhage	Yes	21(31.3)	8(11.9)	**0.006** Chi-square values = 7.43 RR = 0.49, CI95% (0.26, 0.90)
	Mean diff= -2.38, CI95% (-4.82, 0.04)							
Third -day- oxygen index (%)	4.87 ± 4.25	3.69 ± 4.13	0.111 T value=-1.60		No	46(68.7)	59(88.1)	
	Mean diff=-1.17, CI 95% (-2.62, 0.27)							
First -day- Blood Sugar (mg/dL)	86.93 ± 20.78	84.73 ± 21.25	0.547 T value=-0.60	Pneumothorax	Yes	6 [9]	7(10.4)	0.770 Chi-square values = 0.08 RR = 1.08, CI95% (0.63, 1.85)
	Mean diff=- 2.19, CI 95% (-9.37, 4.98)							
Third -day- Blood Sugar (mg/dL)	98.18 ± 21.46	95.27 ± 17.06	0.175 T value=-0.86		No	61(91)	60(89.6)	
	Mean diff=-2.91, CI 95% (-9.57, 3.74)							
First -day- Mean Blood Pressure (mm Hg)	36.04 ± 7.78	38.07 ± 4.95	**0.010** T value = 2.41	Intraventricular Hemorrhage	Yes	16(23.9)	9(13.4)	0.121 Chi-square values = 2.41 RR = 0.67, CI95% (0.39, 1.17)
	Mean diff = 2.03, CI95% (0.36, 3.69)							
Third -day- Mean Blood Pressure (mm Hg)	38.17 ± 4.33	39.52 ± 4.04	0.064 T value = 1.86		No	51(76.1)	58(86.6)	
	Mean diff = 1.35, CI 95% (-0.08, 2.79)							
* Independent t-test, **chi-square test, ***Risk Ratio (RR) † Primary Outcome				Second Dose Surfactant	Yes	41(61.2)	13(19.4)	**0.001** Chi-square values = 24.31 RR = 0.35, CI 95% (0.21, 0. 58)
					No	26(38.8)	54(80.6)	

### The secondary outcomes result

The comparison of the frequency of pulmonary hemorrhage, second dose surfactant, mean of mechanical ventilation use days, and blood pressure between the two groups revealed a significant difference. The surfactant and budesonide combination group showed better outcomes in terms of these factors compared to the group receiving surfactant alone(*p* < 0.05). There was no significant difference between the two groups in terms of other variables (*p* > 0.05). The risk of having Pulmonary Hemorrhage and needs for second dose of surfactant with treatment were 49% and 35% of the risk in control group respectively (Table 2). In the intervention group, the duration of oxygen therapy was ranged between 1 and 9 days, while in the control group, was 1–14 days.

### Subgroup analysis result

In the study, the researchers examined the primary and secondary outcomes separately for two subgroups. The first subgroup was based on birth weight, dividing the participants into three groups: those with birth weight less than 1500 gr. 1500–2000 gr, and 2000–2500 gr birth weight. Similarly, the second subgroup was based on gestational age, dividing the participants into three groups: gestational age less than 30 weeks, 30–34 weeks, and 34–37 weeks. By analyzing the outcomes in these subgroups, the researchers aimed to investigate whether birth weight and gestational age had any impact on the primary and secondary outcomes of the study. This analysis helps to provide a more comprehensive understanding of the results and their implications for different subpopulations.

The subgroup results showed that gestational age had a more significant effect on mortality rate than birth weight. Specifically, administering surfactant with budesonide to infants under 30 weeks of age significantly reduced the number of deaths compared to using surfactant alone (5 vs. 17). Similar positive effects were observed for the occurrence of Pulmonary Hemorrhage (7 vs. 18). Additionally, the combination of surfactant with budesonide also reduced the need for a second dose of surfactant in infants under 34 weeks of age. While there was no difference in the frequency of BPD in the subgroups (Table 3).

**Table 3:** the comparison of frequency of primary and secondary outcome between two groups in term of birth weight and gestational age subgroups

Birth weight	Variables		Surfactant(*n* = 67)	Surfactant + Budesonide (*n* = 67)	*P* value	Gestational age	Surfactant(*n* = 67)	Surfactant + Budesonide (*n* = 67)	*P* value
			Frequency (%)	Frequency (%)			Frequency (%)	Frequency (%)	
< 1500 gr N _G1_= 30 people N _G2_=40 people	**Death**	Yes	17(42.5)	9(30)	0.284 Chi-square values = 1.14	< 30 weeks N _G1_= 24 people N _G2_=16	17(70.8)	5(31.3)	**0.014** Chi-square values = 6.07
			**RR = 0.72, CI 95% (0.39, 1.33)				RR = 0.37, CI 95% (0.15, 0.87)		
	BPD	Yes	10(25)	8(26.7)	0.875 Chi-square values = 0.02		5(20.8)	7(43.8)	0.166 Chi-square values = 2.40
			RR = 1.05, CI 95% (0.57, 1.92)				RR = 1.81, CI 95% (0.88, 3.72)		
	**Pulmonary Hemorrhage**	Yes	18(45)	7(23.3)	0.061 Chi-square values = 3.50		16(66.7)	3(18.8)	**0.003** Chi-square values = 8.83
			RR = 0.54, CI 95% (0.27, 1.09)						
							RR = 0.25, CI 95% (0.08, 0.76)		
	Pneumothorax	Yes	5(12.5)	7(23.3)	0.234 Chi-square values = 1.41		5(20.8)	4(25)	1 Chi-square values = 0.09
			RR = 1.47, CI 95% (0.82, 2.61)						
							RR = 1.14, CI 95% (0.48, 2.69)		
	Intraventricular Hemorrhage	Yes	14(35)	8(26.7)	0.457 Chi-square values = 0.55		10(41.7)	7(43.8)	0.896 Chi-square values = 0.01
			RR = 0.79, CI 95% (0.42, 1.49)						
							RR = 1.05, CI 95% (0.49, 2.25)		
	**Second Dose Surfactant**	Yes	35(87.5)	9(30)	**0.001** Chi-square values = 24.27		23(95.8)	5(31.3)	**0.001** Chi-square values = 19.06
			RR = 0.25, CI 95% (0.13, 0.46)						
							RR = 0.19, CI 95% (0.08, 0.43)		
1500–2000 gr N _G1_= 22 people N _G2_=13 people	Death	Yes	2(15.4)	1(4.5)	0.541 Chi-square values = 1.22	people30-34 weeks N _G1_= 39 people N _G2_=32 people	2(6.3)	4(10.3)	0.683 Chi-square values = 0.36
			RR = 0.50, CI 95% (0.10, 2.56)						
							RR = 1.23, CI 95% (0.67, 2.27)		
	BPD	Yes	0	0	-		5(15.6)	1(2.6)	0.084 Chi-square values = 3.87
							RR = 0.28, CI 95% (0.04, 1.72)		
	Pulmonary Hemorrhage	Yes	3(23.1)	1(4.5)	0.134 Chi-square values = 2.77		5(15.6)	4(10.3	0.722 Chi-square values = 0.45
			RR = 0.36, CI 95% (0.06, 2.05)						
							RR = 0.78, CI 95% (0.36, 1.68)		
	Pneumothorax	Yes	1(7.7)	0	0.371 Chi-square values = 1.74		1(3.1)	3(7.7)	0.746 Chi-square values = 0.69
							RR = 1.39, CI 95% (0.76, 2.56)		
	Intraventricular Hemorrhage	Yes	2(15.4)	1(4.5)	0.541 Chi-square values = 1.22		10(41.7)	7(43.8)	0.196 Chi-square values = 3.26
			RR = 0.50, CI 95% (0.50, 2.56)				RR = 0.42, CI 95% (0.12, 1.42)		
	**Second Dose Surfactant**	Yes	5(38.5)	2(9.1)	0.075 Chi-square values = 4.40		23(95.8)	5(31.3)	**0.001** Chi-square values = 11.43
			RR = 0.40, CI 95% (0.12, 1.32)				RR = 0.37, CI 95% (0.18, 0.77)		
2000–2500 gr N _G1_= 15 people N _G2_=14 people	Death	Yes	0	0	0	34–37 weeks N _G1_= 12 people N _G2_=11 people	0	1(8.3)	1 Chi-square values = 0.95
	BPD	Yes	0	0	0		0	0	-
	Pulmonary Hemorrhage	Yes	0	0	0		0	1(8.3)	1 Chi-square values = 0.95
	Pneumothorax	Yes	0	0	0		0	0	-
	Intraventricular Hemorrhage	Yes	0	0	0		0	0	-
	Second Dose Surfactant	Yes	1(7.1)	2(13.3)	1 Chi-square values = 0.29		1(19.1)	2(16.7)	1 Chi-square values = 0.29
			RR = 1.33, CI 95% (0.54, 3.23)						
							RR = 1.33, CI 95% (0.53, 3.32)		

*chi-square test and fisher exact test, ** Risk Ratio (RR)

Furthermore, within the group of infants who received both surfactant and budesonide, those who were under 30 weeks of age and weighed less than 1500 g showed better outcomes in terms of their oxygen index, mean blood pressure and MAP compared to other subgroups (*p* < 0.05) (Table 4).

**Table 4:** the comparison of mean (SD) secondary outcome between two groups in term of birth weight and gestational age subgroups

Birth weight subgroup	Variables	Surfactant Group (*n* = 67)	Surfactant + Budesonide Group (*n* = 67)	*P* value*	Gestational age subgroup	Surfactant Group (*n* = 67)	Surfactant + Budesonide Group (*n* = 67)	*P* value*
		Mean (SD)	Mean (SD)			Mean (SD)	Mean (SD)	
< 1500 gr N _G1_= 30 people N _G2_=40 people	First -day- MAP (mmHg)	17.53 ± 1.30	16.57 ± 2.37	0.089 F = 4.66	< 30 weeks N _G1_= 24 people N _G2_=16 people	17.79 ± 0.93	16.88 ± 2.36	0.345 F = 2.95
	Third -day – MAP (mmHg)	13.67 ± 2.84	9.21 ± 3.45	**0.001** F = 34.14		14.65 ± 2.19	9.80 ± 3.61	**0.001** F = 26.75
	First -day- oxygen index (%)	18.60 ± 6.11	16.10 ± 0.8.39	0.090 F = 2.08		19.63 ± 5.32	18.06 ± 9.04	0.359 F = 0.47
	Third -day- oxygen index (%)	6.71 ± 4.39	5.26 ± 4.48	**0.045** F = 1.64		8.45 ± 4.11	5.47 ± 4.65	**0.010** F = 4.23
	First -day- Blood Sugar (mg/dL)	85.50 ± 22.24	78.53 ± 19.33	0.175 F = 1.87		87.54 ± 19.21	78.25 ± 18.51	0.137 F = 2.31
	Third -day- Blood Sugar(mg/dL)	97.46 ± 24.14	93.80 ± 18.23	0.491 F = 0.47		97.17 ± 17.77	96.31 ± 18.01	0.883 F = 0.02
	First -day- Mean Blood Pressure (mmHg)	33.45 ± 2.74	34.90 ± 4.54	0.058 F = 2.75		31.88 ± 1.60	33.13 ± 4.69	0.700 F = 1.46
	Third -day- Mean Blood Pressure (mmHg)	35.72 ± 2.61	36.70 ± 2.81	**0.046** F = 2.25		34.91 ± 2.13	35.69 ± 2.85	0.263 F = 0.94
1500–2000 gr N _G1_= 22 people N _G2_=13 people	First -day- MAP (mmHg)	16.16 ± 1.68	14.50 ± 2.26	0.062 F = 5.22	30–34 weeks N _G1_= 39 people N _G2_=32 people	16.47 ± 1.80	14.95 ± 2.38	**0.003** F = 8.87
	Third -day – MAP (mmHg)	8.38 ± 4.07	6.77 ± 1.95	0.853 F = 2.51		10.09 ± 4.05	7.18 ± 2.43	**0.012** F = 14.06
	First -day- oxygen index (%)	12.15 ± 6.44	10.41 ± 4.44	0.647 F = 0.90		14.16 ± 7.14	11.38 ± 5.43	0.175 F = 3.44
	Third -day- oxygen index (%)	3 ± 3.10	2.90 ± 3.85	0.749 F = 0.01		3.44 ± 3.37	3.34 ± 4.16	0.691 F = 0.01
	First -day- Blood Sugar (mg/dL)	93.38 ± 18.62	88.0 ± 20.41	0.442 F = 0.60		90.22 ± 22.08	86 ± 21.24	0.416 F = 0.66
	Third -day- Blood Sugar (mg/dL)	99.0 ± 17.21	95.32 ± 17.75	0.553 F = 0.35		100.88 ± 24.86	94.10 ± 17.48	0.183 F = 1.80
	First -day- Mean Blood Pressure (mmHg)	37.31 ± 4.92	38.73 ± 2.66	0.578 F = 1.23		37.38 ± 4.12	38.44 ± 3.21	0.207 F = 1.48
	Third -day- Mean Blood Pressure (mmHg)	39.38 ± 3.97	40.18 ± 3	0.353 F = 0.45		38.75 ± 3.70	39.87 ± 3.92	0.122 F = 1.81
2000–2500 gr N _G1_= 15 people N _G2_=14 people	First -day- MAP (mmHg)	15.36 ± 1.28	14.73 ± 1.98	0.451 F = 0.99	34–37 weeks N _G1_= 12 people N _G2_=11 people	15.64 ± 1.21	15.33 ± 2.10	0.786 F = 0.17
	Third -day – MAP (mmHg)	6.21 ± 0.58	6.27 ± 0.59	0.621 F = 0.05		6.27 ± 0.65	6.92 ± 1.98	0.397 F = 1.06
	First -day- oxygen index (%)	7.79 ± 3.47	9.27 ± 3.24	0.186 F = 1.41		7.91 ± 3.70	9.83 ± 4.93	0.487 F = 1.10
	Third -day- oxygen index (%)	1.61 ± 0.59	1.83 ± 0.62	0.505 F = 1.01		1.86 ± 0.50	2.64 ± 2.72	0.976 F = 0.87
	First -day- Blood Sugar	85.0 ± 18.36	92.33 ± 23.84	0.364 F = 0.85		76 ± 17.97	89.25 ± 24.39	0.156 F = 2.16
	Third -day- Blood Sugar (mg/dL)	99.43 ± 17.93	98.13 ± 14.08	0.830 F = 0.04		92.45 ± 17.93	97.67 ± 15.39	0.462 F = 0.56
	First -day- Mean Blood Pressure (mmHg)	42.29 ± 2.61	43.47 ± 2.97	0.290 F = 1.28		41.27 ± 3.85	43.50 ± 3.66	0.235 F = 2.02
	Third -day- Mean Blood Pressure (mmHg)	43.86 ± 2.21	44.20 ± 2.46	0.561 F = 0.15		43.27 ± 3.90	43.50 ± 3.15	0.928 F = 0.02

* Analysis of variance (ANOVA)

The Analysis of covariance (ANCOVA) showed that the comparison of the mean score of third-day MAP was statistically significant differences between two groups after adjusting the first-day scores. The effect of intervention in improving third-day MAP was 8% (Eta = 0.085). While, the effect of intervention on the other secondary variables was not significant (*p* > 0.05) (Table 5).

**Table 5:** the comparison of mean (SD) secondary outcome between two groups by adjusting the baseline variables**

	Sum of Squares	df	Mean Square	F	*P* value*	Partial Eta Squared
Third-Day MAP	94.715	1	94.715	11.995	0.001	0.085
Third-Day OI	0.188	1	0.188	0.027	0.870	0.000
Third-Day Blood Sugar	130.376	1	130.376	0.442	0.507	0.003
Third-Day Mean Blood Pressure	0.109	1	0.109	0.021	0.885	0.000

* Analysis of covariance (ANCOVA)

** The baseline variables included the first day MAP,” “first-day oxygen index,” “first-day blood sugar,” and “first day mean blood pressure

## Discussion

This study aimed to compare the impact of administering both intratracheal surfactant and budesonide versus surfactant alone on the mortality rate and BPD in preterm infants with respiratory distress syndrome. The overall analysis did not reveal a significant difference in mortality rate and BPD between the two groups. However, subgroup analysis showed that gestational age had a significant influence on mortality rate. Specifically, the combination of surfactant and budesonide significantly reduced the number of deaths in infants under 30 weeks of age compared to surfactant alone. The difference in BPD between subgroups was not significant. In a systematic review published in 2023, the findings suggested that three randomized clinical studies reported a reduction in the incidence of BPD and death when using intratracheal surfactant with budesonide compared to surfactant alone. However, two observational studies and one clinical trial did not find any statistically significant differences between the groups in terms of BPD and death [7]. The included studies focused on short-term outcomes in infants weighing less than 1500 g or born before 30 weeks. The findings of our study show that infants under 30 weeks with respiratory distress benefit from the combination of surfactant and budesonide in preventing hospital mortality.

In a 2016 clinical trial conducted by Yeh et al., the effect of intratracheal administration of budesonide plus surfactant was evaluated in 265 very-low-birth-weight infants with severe respiratory distress syndrome. The trial took place in the United States and Taiwan and included infants who required mechanical ventilation and high levels of inspired oxygen shortly after birth. The results showed that the co-administration of budesonide/surfactant significantly reduced the incidence of BPD and death compared to surfactant alone, without any observed side effects [8]. These findings differ from the overall results obtained in our study. One possible explanation for this discrepancy could be the difference in gestational age of the infants included in the studies. Our study focused on infants below 37 weeks of gestational age. It is important to note that gestational age can be a significant confounding factor in studies involving preterm infants. In our study, the subgroup results showed that gestational age had a more significant effect on mortality rate than birth weight. Specifically, administering surfactant with budesonide to infants under 30 weeks of age significantly reduced the number of deaths compared to using surfactant alone. This finding is similar to Yeh et al.‘s study [8].

In a 2017 study conducted by Venkataraman et al. in Canada, it was concluded that the co-administration of budesonide with intratracheal surfactant was associated with a reduction in the incidence of BPD alone or a concomitant reduction in mortality and BPD in very low birth weight infants [15]. The findings of Venkataraman et al.‘s study differ from our study, where the combination therapy did not show a significant reduction in BPD and mortality. These discrepancies in results could be attributed to various factors such as differences in gestational age or intervention protocols. It is important to consider these factors when interpreting and comparing study findings.

In our study, the comparison of the frequency of pulmonary hemorrhage, second dose surfactant, mean of mechanical ventilation use days, and blood pressure between the two groups revealed a significant difference. The surfactant and budesonide combination group showed better outcomes in terms of these factors compared to the group receiving surfactant alone. While there was no significant difference between the two groups in terms of other variables.

In a pilot study conducted in Taiwan by Kuo et al. in 2010, infants in the intervention group who received intratracheal administration of budesonide/surfactant showed improved pulmonary condition compared to the control group. The intervention group had significantly higher PaO2 (partial pressure of oxygen) and lower oxygen indices on the second and third days of treatment. They also had lower continuous positive airway pressure (CPAP) requirements. The study compared both groups in terms of mortality, physical growth, blood pressure, blood sugar levels, and the number of times surfactant needed to be administered. The results indicated that the budesonide/surfactant intervention improved pulmonary condition without any complications [16].

In our study, the blood sugar levels recorded in both groups on the first and third days of treatment did not show any significant differences. However, there was a significant difference in the oxygenation index between the two groups. This suggests that the intratracheal administration of budesonide/surfactant had a positive impact on oxygenation in the study population. A similar finding was reported in the study of Yeh et al.‘s study in terms of pulmonary hemorrhage with, a second dose of surfactant [8].

The subgroup results showed that within the group of infants who received both surfactant and budesonide, those who were under 30 weeks of age and weighed less than 1500 g showed better outcomes in terms of their oxygen index, mean blood pressure, and MAP compared to other subgroups. The mean scores of third-day MAP were statistically significant differences between two groups after adjusting the first-day scores. In a study conducted by Kothe et al. in 2019 in the United States, the efficacy of combining surfactant and budesonide was evaluated. Infants with RDS received budesonide (0.25 mg/kg) in combination with surfactant (4 ml/kg), and their outcomes were compared with a retrospective cohort of infants from 2013 to 2016 who received surfactant alone. The study found that there was no significant difference in the rates of death or BPD between the two groups. Additionally, secondary morbidities such as necrotizing enterocolitis, intraventricular hemorrhage, premature retinopathy, and sepsis were similar in both groups [9]. These findings are consistent with the results of our intervention study.

in the 2020 study by Heo et al., 34 infants weighing less than 1,500 g with severe RDS were included. The study compared the outcomes of infants receiving combination therapy (budesonide with surfactant) with those receiving surfactant alone. Although the combination therapy group showed lower rates of surfactant re-administration, shorter ventilation duration, lower mortality, and lower incidence of BPD compared to the surfactant group, these differences were not statistically significant. However, the duration of hospitalization was significantly shorter in the intervention group compared to the control group [17]. In our study, contrary to the findings of the mentioned study, the need for a second dose of surfactant showed a significant difference. This difference may be attributed to various factors such as differences in gestational age or intervention protocols.

### The strengths of our study

The strengths of our study include the analysis of subgroups based on the age and weight of the infants, which provided a clearer understanding of the intervention’s effects. Additionally, the study’s adherence to standard methods for designing and implementing the intervention enhances the reliability of the findings.

### Limitations

There are limitations to consider. One limitation is that the follow-up period was limited to the duration of hospitalization until discharge, and long-term outcomes after discharge were not investigated. This limits the ability to assess the sustained effects of the intervention on outcomes beyond the hospital stay. Another limitation is the sample size, which may not have been sufficient to fully address the secondary outcomes of the study. Conducting further studies with larger sample sizes and longer follow-up periods is recommended to obtain more robust conclusions. This study was conducted in the form of a single-blind methodology. To enhance the validity of future conclusions, it is recommended to conduct a randomized clinical trial with double-blinding. In this study, the details of ABG analysis were not the primary focus or outcome of the study. It was a limitation of the study. To address this limitation, future research could consider conducting a separate analysis specifically focusing on ABG parameters. This additional analysis could provide insights into the respiratory status, acid-base balance, and gas exchange efficiency of the neonates.

## Conclusion

Based on the results, the combination of surfactant and budesonide showed promising results in terms of reducing mortality rate and improving secondary outcomes such as pulmonary haemorrhage, second dose surfactant requirement, and measures of mechanical ventilation use, oxygen index, and blood pressure. However, the overall comparison did not show statistically significant differences in mortality rate or BPD between the two groups. The subgroup analysis revealed that gestational age had a more significant impact on mortality rate than birth weight, with the combination therapy showing significant reductions in mortality and pulmonary haemorrhage in infants under 30 weeks of age. The combination therapy also reduced the need for a second dose of surfactant in infants under 34 weeks of age. Additionally, infants under 30 weeks of age and weighing less than 1500 g had better outcomes in terms of oxygen index, mean blood pressure, and mean arterial pressure when receiving the combination therapy. These findings suggest that the combination therapy of surfactant and budesonide may be beneficial, particularly in preterm infants with lower gestational age and birth weight. However, further studies with larger sample sizes and longer follow-up periods are needed to confirm these results and assess long-term outcomes.

## Data Availability

The dataset used in the present study is available from the corresponding author upon reasonable request.

## Abbreviations

(BPD): Bronchopulmonary Dysplasia
(RDS): Respiratory Distress Syndrome
(MAP): Mean Arterial Pressure
(NVD): Vaginal Delivery
(CS): Cesarean Section Deliveries
(FRC): Functional Residual Capacity
(CPAP): Continuous Positive Airway Pressure
(NICU): Neonatal Intensive Care Unit
(ANOVA): Analysis of variance
(ANCOVA): Analysis of covariance
(PaO2): Partial Pressure of Oxygen
(RR): Risk Ratio
